# Programmed Cell Death May Be Involved in the Seedless Phenotype Formation of Oil Palm

**DOI:** 10.3389/fpls.2022.832017

**Published:** 2022-03-23

**Authors:** Yin Min Htwe, Peng Shi, Dapeng Zhang, Zhiying Li, Yong Xiao, Yaodong Yang, Xintao Lei, Yong Wang

**Affiliations:** ^1^Hainan Key Laboratory of Tropical Oil Crops Biology/Coconut Research Institute of Chinese Academy of Tropical Agricultural Sciences, Wenchang, China; ^2^Hainan Key Laboratory for Biosafety Monitoring and Molecular Breeding in Off-Season Reproduction Regions/Sanya Research Institute of Chinese Academy of Tropical Agricultural Sciences, Sanya, China; ^3^Hainan Yazhou Bay Seed Laboratory, Sanya, China; ^4^Tropical Crops Genetic Resources Institute of Chinese Academy of Tropical Agricultural Sciences, Haikou, China

**Keywords:** seedless, PCD, MADS-box, S-RNase, 4-coumarate-CoA ligase

## Abstract

Oil palm (*Elaeis guineensis* Jacq.) is a well-known vegetable oil-yielding crop. Seedlessness is one of the most prominent traits in oil palm due to its low processing costs and high oil content. Nevertheless, an extensive study on molecular mechanisms regulating seedless phenotype formation in oil palm is very limited so far. In this study, stigma, style, and ovary from seedless and seeded (Tenera and Pisifera) oil palm trees were used to investigate the possible mechanism. Results showed that non-pollination resulted in no fruits, and self- and cross-pollinations resulted in seedless fruits, while boron treatment had no effect on seedless phenotype formation, implying that seedless trees have incomplete self and outcrossing incompatibility. Furthermore, the transcriptome data analysis highlighted eight programmed cell death (PCD) genes and three groups of PCD-related genes: 4-coumarate-CoA ligase (4CL), S-RNase, and MADS-box. The majority of these genes were significantly up-regulated in the stigma and style of Seedless palm trees compared to Tenera and Pisifera. In addition, the co-expression network analysis confirmed the significant correlation among these genes. Moreover, two simple sequence repeats (SSR) markers (S41 and S44) were developed to identify the seedless phenotype. The up-regulation of 4CL and MADS-box TFs activated the expression of PCD genes; on the other hand, S-RNase resulted in pollen tube RNA degradation and triggered PCD. While the link between PCD and seedless phenotype formation in oil palm has not been extensively studied to date, these findings suggest a role of PCD in pollen tube lethality, leading to double fertilization failure and the seedless phenotype.

## Introduction

Oil palm (*Elaeis guineensis* Jacq.) is the world’s largest source of vegetable oil, which contributes 33% of vegetable oil and 45% of edible oil production all over the world (Wang et al., 2018a,b). With the fast-growing population, the development of high oil-yielding genetic material has become highly desirable to cope with the increased demands of edible oil (Wang et al., 2018a; Romero et al., 2021). In oil palm, the mesocarp and kernel of the fruit are the richest sources of oil, and the majority of the fruit in seedless oil palm is occupied by oil-storing mesocarp (Somyong et al., 2018; Shi et al., 2019). Hence, seedless phenotype becomes a highly appreciated trait in oil palm to improve oil yield.

Seedless fruits can result mainly from (1) parthenocarpy, in which the fruits develop without fertilization, or from (2) stenospermy, in which pollination and fertilization occur, but embryos degenerate before the completion of seed formation (Vardi et al., 2008; Lora et al., 2011; Somyong et al., 2018). Besides, boron deficiency can affect pollen germination, pollen tube growth, fruit set, and seed formation, thereby inhibiting reproductive growth (Alva et al., 2015). Previous studies on seedless phenotype were principally focused on other species, such as Arabidopsis (Goetz et al., 2007), tomato (Klap et al., 2017; Takisawa et al., 2018; Molesini et al., 2020), grapevine (Malabarba et al., 2017; di Rienzo et al., 2021; Li et al., 2021), citrus (Zhang et al., 2018; Liang et al., 2020), eggplant (Chen et al., 2017), sugar apple (Lora et al., 2011), and pear (Wang et al., 2021a,b).

Phenylpropanoid biosynthesis pathway is an important pathway for plant development, including somatic embryogenesis (Zhang et al., 2021) and floral organs (Zhang et al., 2018). In rice, 4-coumarate-CoA ligase (4CL)-like gene, *OsAAE3*, negatively regulates floral development. The over-expression of *OsAAE3* in rice results in programmed cell death (PCD) and, consequently decreases pollen fertility (Liu et al., 2017a). Seedlessness in citrus has been reported to be associated with ovule sterility or self-incompatibility (SI; Montalt et al., 2021). SI system prevents self-fertilization by rejecting self-pollen in the style of flowering plants (Claessen et al., 2019). In most cases, the SI system in flowering plants is genetically controlled by the S-locus, which includes at least two tightly linked polymorphic genes: male- and female-specificity determinants (pollen-S, pistil-S) (Liang et al., 2020). It has been proposed that two tightly linked polymorphic genes at the S-locus [S receptor kinase gene (*SRK*) and S-locus glycoprotein gene (*SLG*)] cooperatively function in SI response (Takasaki et al., 2000). Pistil-S gene encodes S-RNase, which causes pollen tube lethality by catalyzing RNA degradation (Claessen et al., 2019). A previous study in pear reported that self-pollen rejection by S-RNase triggered PCD (Wang et al., 2009). Singh et al. (2013) reported that *SHELL* gene is responsible for endocarp development, which differentiates three fruit forms: Dura, Tenera, and Pisifera in oil palm. The authors found two independent mutations in MADS-box transcription factor *SHELL*, a homolog of the MADS-box gene *SEEDSTICK* (also known as *AGAMOUS-Like* 11, *AGL11*), which acts on the ovule identity and seed development in Arabidopsis (Singh et al., 2013, 2020). In addition, MADS-box genes have also been reported to be implicated in pollen fertilization and the development of seedless fruits (Klap et al., 2017; Takisawa et al., 2018; Tang et al., 2019; Wei et al., 2019; Molesini et al., 2020). MADS-box gene *AGL11* has been proposed as the key gene responsible for triggering the seedless phenotype (Ocarez and Mejía, 2016). An earlier study in rice (Yang et al., 2012) suggests that *OsMADS29* affects the seed development by regulating cell degeneration through PCD-related genes. In pear, PCD mechanism also lead to ovule abortion, resulting in seedless fruits (Wang et al., 2021b).

In oil palm, despite the appreciation of phytohormones treatment for inducing seedless fruits (Somyong et al., 2018; Romero et al., 2021), the knowledge of possible molecular mechanisms controlling seedlessness is not clear so far. Thus, to better understand the mechanism, we performed self-, cross-, and non-pollinations as well as the exogenous application of boron to elucidate their effects on oil palm seedlessness. Thereafter, transcriptomics (RNA-seq) analysis was performed to further investigate the mechanisms. In addition, our study provided markers that could be useful for early selection of oil palm seedless plants, which will be of benefit to accelerate oil palm breeding process.

## Materials and Methods

### Pollination and Boron Treatment Analysis

Seedless, Tenera, and Pisifera oil palm trees were conserved in the national tropical palm germplasm resource nursery in Wenchang, Hainan, China (19°31′40′′–19°31′58′′N, 110°45′54′′–110°46′4′′E. Trees aged 10 years were selected in the present study.

To test whether pollination has effects on seedless fruit development or not, we performed self-, cross-, and non-pollination treatments. Female inflorescences were bagged before bracts split. For non-pollination treatment, bags were taken off 14 days later; while pollination treatments were conducted 7 days after bracts split and bags were taken off 14 days later. To further investigate whether seedless pollen is able to germinate, pollen germination was examined using *in vitro* germination method, as described previously (Wang et al., 2018b). To further determine if boron treatment can affect seedless fruit development in oil palm, different concentrations of boron (1.25, 2.5, and 5 g/L) were applied on opened female inflorescences three times (once per day). No boron treatment was considered as the control. The investigation of fruit development was carried out 180 days after boron application. All pollination and boron treatments were performed in three replicates.

### DNA Extraction

Total genomic DNA from the leaves of different fruit types was extracted using Rapid Plant Genomic DNA isolation Kit (Sangon Biotech, Shanghai, China), according to the manufacturer’s protocol. The mean (three replicates) DNA concentration (ng μl^–1^) and the optical density (OD) ratios 260/280 were calculated by measuring the OD at wavelengths of 260 and 280 nm using DS-11 Spectrophotometer (DeNovix). The DNA was further diluted to a working concentration of 50 ng μl^–1^ with Tris-EDTA (TE) buffer and used for simple sequence repeats (SSR) analysis.

### RNA Extraction

**FIGURE 1:**
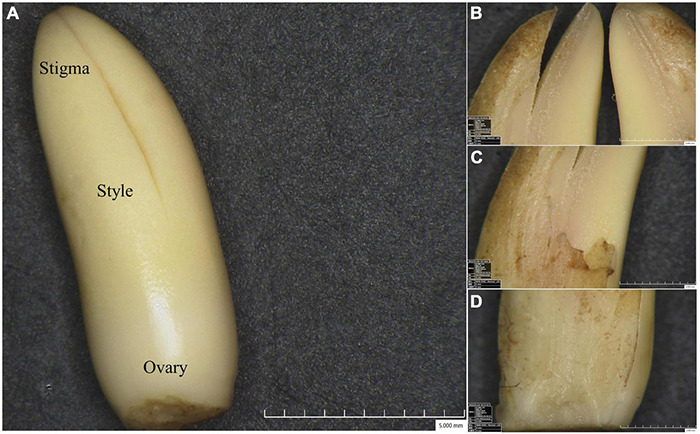
Floral tissues viewed under digital microscope. **(A)** The pistil showing stigma (S), style (ST), and ovary (O). Detailed sectional views of stigma **(B)**, style **(C)**, and ovary **(D)**.

Female inflorescences from Pisifera (P), Tenera (T), and Seedless (S) palm trees were harvested before bracts split. Various floral tissues, including stigma (S), style (ST), and ovary (O) were then collected (Figure 1). These tissues were immediately frozen in liquid nitrogen and stored at −80°C.

Total RNA was extracted using TRIzol reagent kit (Invitrogen, Carlsbad, CA, United States) according to manufacturer’s instruction, and NanoDrop ND-2000 spectrophotometer (NanoDrop, United States) was used to determine total RNA concentration and purity. RNA quality was assessed on an Agilent 2100 Bioanalyzer (Agilent Technologies, Palo Alto, CA, United States) and checked using RNase-free agarose gel electrophoresis.

### Transcriptome Sequencing

After total RNA was extracted, eukaryotic mRNA was enriched by Oligo(dT) beads, while prokaryotic mRNA was enriched by removing rRNA by Ribo-Zero™ Magnetic Kit (Epicentre, Madison, WI, United States). Then the enriched mRNA was fragmented into short fragments using fragmentation buffer and reverse transcripted into complementary DNA (cDNA) with random primers. Second-strand cDNA was synthesized by DNA polymerase I, RNase H, deoxynucleoside triphosphate (dNTP), and buffer. Then the cDNA fragments were purified with QIAquick PCR Extraction Kit (Qiagen, Venlo, Netherlands), end-repaired, poly(A) added, and ligated to Illumina sequencing adapters. The ligation products were size-selected by agarose gel electrophoresis, PCR amplified, and sequenced using Illumina HiSeq 2500 by Gene Denovo Biotechnology Co. (Guangzhou, China).

After sequencing, raw reads containing adapters or low-quality bases were further filtered, and clean reads were used for downstream bioinformatics analysis. The clean reads were mapped to a reference genome using HISAT2. 2.4 (Kim et al., 2015) with “-rna-strandness RF” and other parameters set as a default. The RNAs differential expression analysis was performed by DESeq2 (Love et al., 2014) software between two groups, and edgeR (Robinson et al., 2009) between two samples. The genes/transcripts with the parameter of false discovery rate (FDR) < 0.05 and absolute fold change ≥ 2 were considered differentially expressed genes (DEGs)/transcripts. Then, Gene Ontology (GO) classification and Kyoto Encyclopedia of Genes and Genomes (KEGG) pathway enrichment of DEGs were analyzed to evaluate their biological functions.

### Primer Design

To design SSR primers, nucleotide sequences of previously reported six known S-RNases were downloaded from National Center for Biotechnology Information (NCBI) (HE805271.1 and AJ315593.1 from Antirrhinum; D63887.1 and AB568389.1 from Solanaceae; and FJ543097.1 and AF327223.1 from Rosaceae) (Liang et al., 2020) and blast against oil palm whole genome to detect sequence similarity. Among them, HE805271.1 showed 84.85% (*E*-value 3e−11) similarity with KE607205.1, 84.85% (*E*-value 2e−11) with ASJS01098507.1, and 95.45% (*E*-value 4e−10) with CM002081.1. Seven primers were designed from the whole genome shotgun sequence KE607205.1 (GeneBank). One primer was designed from the whole genome shotgun sequence ASJS01098507.1 (GeneBank). Twelve primers were designed from the whole genome shotgun sequence CM002081.1 (GeneBank). Primer pairs were designed around SSR motifs (Table 1) using the WebSat online software.^1^ The default setting for all repeats was adjusted to a minimum of three repeats, and primer pairs were synthesized by BGI Tech (China).

**TABLE 1:** Details of SSR primers used for the study.

Serial no.	Primer name	GeneBank ID	SSR motif	Sequence (5′–3′)	Product size (bp)
1	S-1	KE607205.1	(A)11	F-AAAAGGGCAAATCAACGTAGTG R-GACGGTTTCACCAAATGTATCA	368
2	S-2	KE607205.1	(A)10	F-TACTCCACCCACTAGCTGACAA R-TTCCTGTTTCTCCACTGTTTGA	161
3	S-3	KE607205.1	(AATTT)3	F-GGATCAGTGCCAGGTTCATTA R-TCCTATAACTGGATGGTCTGGTG	383
4	S-4	KE607205.1	(CTT)4	F-TACTGCGTTGATCCTTCTTTGA R-CCTTATCTTGCCATGTTTGGAT	393
5	S-5	KE607205.1	(AG)5	F-CCACCTTTTGAGACCTAGAAGC R-TTAGCCAAGGAAGTTTTGTGGT	341
6	S-6	KE607205.1	(TATT)3	F-GTTCGCATTGAAGATTGATCC R-TATGACGTGGCTCTGTTACCAC	247
7	S-7	KE607205.1	(CT)5	F-GTCAAGGAGAAGAAGGGACCTC R-TAGGCTGACCAACACCTGAAAT	259
8	S-12	ASJS01098507.1	(TATT)3	F-AGGCCAAAGAGGTAAATGTTCA R-CATGGCTGTTCTTCTTCTTCCT	359
9	S-34	CM002081.1	(TTATT)2.2	F-CGCCTTTTCCAAGCATATAAAG R-ATCTCCCCACATCTCTCTCACA	279
10	S-35	CM002081.1	(TTA)4	F-TTCCTAACGCTAATTGCCTCAT R-ATACCTCCTGTTGTCTCCTCCA	328
11	S-36	CM002081.1	(A)12	F-AAACAGAGCGAACACGACTTTT R-ATGAGGCAATTAGCGTTAGGAA	342
12	S-37	CM002081.1	(ATAC)2.75	F-ATATACTTGGGCATGGGATCT R-CTTAGGATAGTGCCTCGCAT	387
13	S-38	CM002081.1	(AGA)4	F-TGTTTTGGATTGATCTGAGTGG R-CACAGCCTCGTTGAAAAGATAG	309
14	S-39	CM002081.1	(A)11	F-TGATTAAGTTGGAGGGGAGAAA R-TTCATACCTCTTGACGCAAAAC	389
15	S-40	CM002081.1	(AAT)4	F-ATGACCTCTCGATTTTACCCAA R-GGCCAAACTAGATAAATGCACA	371
16	S-41	CM002081.1	(T)8	F-TTAAACGATGAATCCGAGCC R-CGTGAAGGAGGAGGAAGAAAA	392
17	S-42	CM002081.1	(TG)5.5	F-TAGGGCATGTTTGGTATTCCTT R-CACACACACACACACACACAGA	287
18	S-43	CM002081.1	(GA)9	F-TATCATTGGATCTCTGGTGTGG R-CCCAGCAAATAACCTTGAGTTC	247
19	S-44	CM002081.1	(AT)19	F-TGGTGACGAACATTACCTTGAG R-TCTCCTGCCCTGATTCTTTAAC	295
20	S-45	CM002081.1	(ATTTT)3	F-GAAGAAAGAAAGAGAGGTCATCAAC R-GATGTTGTCGTTAGAATATCGTGC	280

The S-locus genes such as S-RNase, SRK, and SLG were selected to design primers for RT-qPCR analysis. Sequences that have a significant similarity with six known S-RNases were detected using BLASTN program from the NCBI. Genes up-regulated in seedless pistil were selected. Gene-specific primers were designed based on transcriptome sequences using the NCBI Primer-BLAST. Primers for RT-qPCR were designed using Primer-BLAST, and the primers that showed a single band of correct size were selected for RT-qPCR analysis (Table 2).

**TABLE 2:** Primer sequences and amplicon length used for RT-qPCR analysis.

Primer name	Gene ID	Primer sequences (5′–3′)	Product size (bp)
S20-RNase	LOC105044992	F-GGCAAGGCCAAGAAAACTGG R-GCCACCTGTAACCGTCTTCA	124
SRK-16	LOC105037373	F-ATCAGGACACGACCATGCTG R-GCAACCCCTTGTAGACTGCT	126
SLG	LOC105037350	F-CGAGACTGGAGCGTTTCAAG R-TTGAGGAGACTTGGGACTGA	181

### PCR Amplification

The PCR amplifications were performed in a total reaction mixture of 20 μl containing: 200 ng of genomic DNA (1 μl), 0.4 μl dNTPs, 2 μl 10× RealTimes buffer containing MgCl_2_, 2 μl RealTimes *Taq* DNA polymerase (5 U μl^–1^), 0.8 μl forward primer (10 μM), 0.8 μl reverse primer (10 μM), and 13 μl sterile ddH_2_O. Amplification conditions for each primer pair was optimized using a touchdown gradient approach under the following conditions: initial denaturation at 94°C for 5 min; denaturation for 30 s, followed by decreasing the annealing temperature by 1°C for each cycle from 65 to 56°C by running 10 cycles; and then the annealing temperature was maintained at 55°C for the next 25 cycles. The annealing time for each cycle was 45 s, elongation time was 1 min at 72°C, and final elongation was at 72°C for 10 min, after which the temperature was maintained at 4°C for infinity. The amplifications were performed in a Biometra Thermal Cycler (Analytik Jena, Germany).

### RT-qPCR Analysis

The plant materials and RNA samples used for RT-qPCR analysis were the same as Illumina sequencing. cDNA from total RNA was prepared using RevertAid™ first strand cDNA synthesis kit (Fermentas, Lithuania) according to the Manufacturer’s protocol. The RT-qPCR was performed on ABI-7900HT (Applied Biosystems, United States) using 2X PowerUp SYBR Green Master Mix (Applied Biosystems, United States) under the following conditions: 95°C for 10 min, followed by 40 cycles at 95°C for 15 s and 60°C for 1 min in 384-well optical plates. Each reaction mixture was 10 μl containing 1 μl of 50 ng/μl cDNA, 5 μl of 2X PowerUp SYBR Green Master Mix, 0.5 μl of each 10 μM forward and reverse primer. The housekeeping gene Act was used as an endogenous control for the normalization of gene expression. The comparative Ct method was used for RT-qPCR normalization, and each sample was detected in three replicates.

### Simple Sequence Repeats Marker Analysis

Dura, Pisifera, Tenera, and Seedless palms were subjected to marker analysis and a total of 20 SSR primers developed in this study were used. Twenty microliters of PCR products were mixed with 1 μl of 6× loading dye and 1 μl was loaded into the well for separation. Separation was carried out on 6% polyacrylamide gel using electrophoresis with DYCZ-20H vertical electrophoresis system (LIUYI, Beijing). The gel image was captured using white light transilluminators. Based on their polymorphisms, two SSR primers were chosen to verify their accuracy for the detection of seedless trait. The accuracy of selected markers for seedless trait selection was further tested using 100 individuals (30 Dura, 30 Pisifera, 30 Tenera, and 10 Seedless palm trees).

### Statistical Analysis

Statistical analyses were performed using IBM SPSS Statistics for Windows, version 26.0. Armonk, NY: IBM Corp. Data were analyzed from the mean fragments per kilobase of transcript per million mapped reads (FPKM) values of three replicates. Error bars represent ± standard deviation. Two-tailed Student’s *t*-test was performed comparing seedless and seeded (Tenera and Pisifera) palm trees. The significance thresholds were set to *p*-value < 0.01. Correlation plots and heatmaps were generated using R (R Core Team, 2018) (version 4.0.3) and RStudio (RStudio Team, 2015) (version 1.4.1103). Cytoscape (Shannon et al., 2003) (version 3.3.0) was used for the generation of a correlation network.

## Results

### Effect of Pollination and Boron on Seedless Phenotype

**FIGURE 2:**
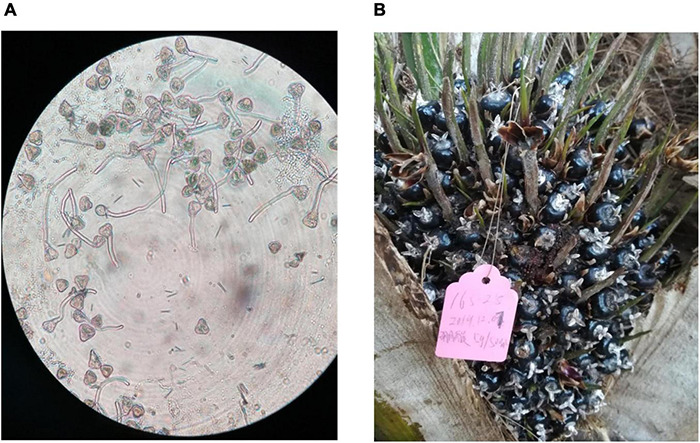
Analysis of the effect of pollination and boron on seedless phenotype. **(A)** Germinated pollen grains from seedless fruit tree. **(B)** Fruits developed from boron-treated female inflorescences.

Pollination results showed that both cross- and self-pollinations resulted in about 30% seedlessness in seedless fruit trees, while no-pollination resulted in no fruits, indicating incomplete self and outcrossing incompatibility. Furthermore, pollens from seedless fruit trees were found to be germinated normally (Figure 2A). Similarly, with untreated control, boron-treated female inflorescences produced normal fruits (Figure 2B). This provides good evidence that both pollination and boron treatment have no effect on seedless phenotype formation, indicating that seedlessness could be controlled by genetic factors.

### Assessment of Transcriptome Sequencing (RNA-seq)

Tenera fruits have normal endocarp and kernel, Pisifera fruits have kernel without endocarp, while Seedless fruits have no endocarp and kernel. Thus, these three fruit forms were selected for comparative analysis to dissect molecular mechanism of seedlessness. Twenty-seven RNA-seq libraries were generated from ovary, stigma, and style of Tenera, Pisifera, and Seedless (TO, TS, TST, PO, PS, PST, SO, SS, and SST) palm trees with three biological replicates. Each library produced over 34 million high-quality clean reads, and the percentage was above 99% among the raw reads (Supplementary Table 1 and Supplementary Figure 1). The expression level of each gene is determined by the fragments per kilobase of transcripts per million fragments mapped (FPKM) method. The results of gene expression and related information for all samples are given in Supplementary Table 2.

### Differentially Expressed Genes Analysis

**FIGURE 3:**
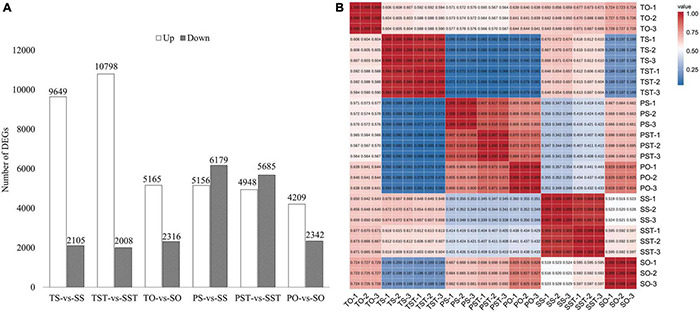
Analysis of DEGs. **(A)** The statistics of DEGs between groups. T, Tenera; P, Pisifera; S, Seedless; S, stigma; ST, style; O, ovary. **(B)** Heatmap of pairwise correlations between samples based on gene expression. Color bars represent the values of Pearson correlation.

The DEGs were identified in (a) TS-vs-SS, (b) TST-vs-SST, (c) TO-vs-SO, (d) PS-vs-SS, (e) PST-vs-SST, and (f) PO-vs-SO. In stigma, 11,754 genes were differentially expressed in Seedless palm trees compared to Tenera, and 11,335 in Seedless palm trees compared to Pisifera. In style, 12,806 genes were differentially expressed in Seedless palm trees compared to Tenera, and 10,633 genes were differentially expressed in Seedless palm trees compared to Pisifera. In ovary, a total of 7,481 genes were differentially expressed in Seedless palm trees compared to Tenera, and 6,551 in Seedless palm trees compared to Pisifera (Figure 3A). Of note, DEGs analysis revealed that there was a greater variation in stigma and style than ovary. Correlation analysis based on gene expression levels showed that the correlations of gene expression levels between the three replicates were high with an average coefficient (*R*) of ≥0.99, suggesting that RNA sequencing generated reliable expression results. The three fruit types (Tenera, Pisifera, and Seedless) are well differentiated from each other. In each fruit type, style and stigma were closer together than ovary (Figure 3B). Together, these results revealed that stigma and style showed similar transcriptome profiles in oil palm seedless fruit formation.

### Metabolic Pathways and Gene Ontology Involved in the Seedless Phenotype

**FIGURE 4:**
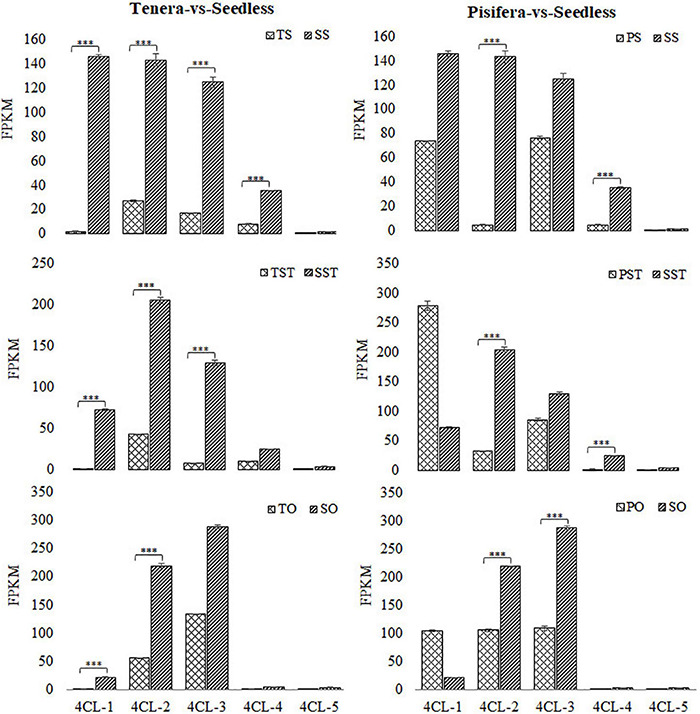
Expression changes of 4-coumarate-CoA ligase (4CL) in Seedless palm trees compared with Tenera and Pisifera in various floral tissues (S, stigma; ST, style; O, ovary). Values shown are means ± SD from three replicates, and asterisks (^***^P ≤ 0.001) indicate a significant difference according to the Student’s *t*-test.

To further reveal the metabolic pathways that are active in seedless oil palm, an analysis of KEGG pathway enrichment was performed. Phenylpropanoid biosynthesis pathway was involved in the Top 20 significantly enriched pathways in all the groups except in TS-vs-SS, in which it is involved in the Top 25 pathways (Supplementary Figure 2 and Supplementary Table 3). Phenylpropanoid biosynthesis pathway has been reported to be involved in the development of floral organs (Zhang et al., 2018). Furthermore, 4CL, which is involved in phenylpropanoid biosynthesis pathway was found to be enriched in pollen development (GO:0009555) and pollen wall assembly (GO:0010208) of the GO biological process (Supplementary Table 4). In rice, overexpression of *OsAAE3*, which encodes a 4CL like protein, can lead to PCD, which contributed to the repressing of floral development and decreased fertility rate of the anther (Liu et al., 2017a; Zhang et al., 2018). To establish the role of 4CL in oil palm seedless phenotype formation, we examined the expression of 4CL encoding genes in floral tissues (stigma, style, and ovary) of seedless and seeded (Tenera and Pisifera) fruits. As shown in Figure 4, most 4CL genes were significantly up-regulated (^***^P ≤ 0.001) in Seedless palm trees compared with Tenera and Pisifera in all the tested tissues.

### Analysis of Programmed Cell Death Genes

**FIGURE 5:**
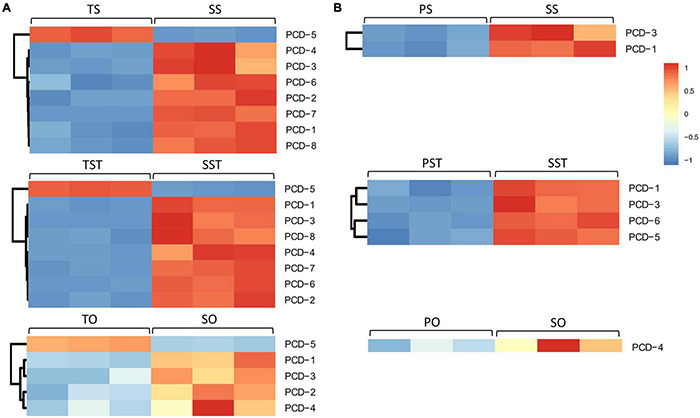
Expression patterns of programmed cell death (PCD) genes in Seedless palm trees compared with **(A)** Tenera and **(B)** Pisifera in various floral tissues (S, stigma; ST, style; O, ovary).

To elucidate the role of PCD in seedless phenotype formation, we analyzed the PCD genes that changed in Seedless palm trees compared to Tenera and Pisifera (Supplementary Table 5). In Tenera-vs-Seedless, a total of eight PCD genes (PCD1–PCD8) were differentially expressed in at least one tested tissue while five PCD genes (PCD1, PCD3, PCD4, PCD5, and PCD6) were differentially expressed in at least one tested tissue in Pisifera-vs-Seedless. Compared with the ovary, more PCD genes were expressed in the stigma and style in both Tenera-vs-Seedless and Pisifera-vs-Seedless. Notably, the expressions of PCD genes, except PCD5, were higher in Seedless palm trees than Tenera and Pisifera (Figure 5).

### Analysis of S-Locus Genes

Self-incompatibility, also known as self-sterility, is a mechanism that rejects their self-pollen and inhibits seed formation in the absence of cross-pollination (Montalt et al., 2021). Molecular studies revealed that genes within the S-locus, including S-RNase, SRK, and SLG, regulate the SI system (Kachroo et al., 2002; Wang et al., 2009; Liang et al., 2020). Besides the SI response, S-RNase also functions as the regulator of PCD (Takayama and Isogai, 2005; Franceschi et al., 2012; Muñoz-Sanz et al., 2020). In this study, the expression profiles of S-locus genes [S-RNase, G-type lectin S-receptor-like serine/threonine-protein kinase (SRK), and S-locus-specific glycoprotein S13-like (SLG)] in stigma, style, and ovary of Tenera-vs-Seedless and Pisifera-vs-Seedless were analyzed to determine their involvement in the formation of seedless phenotype.

**FIGURE 6:**
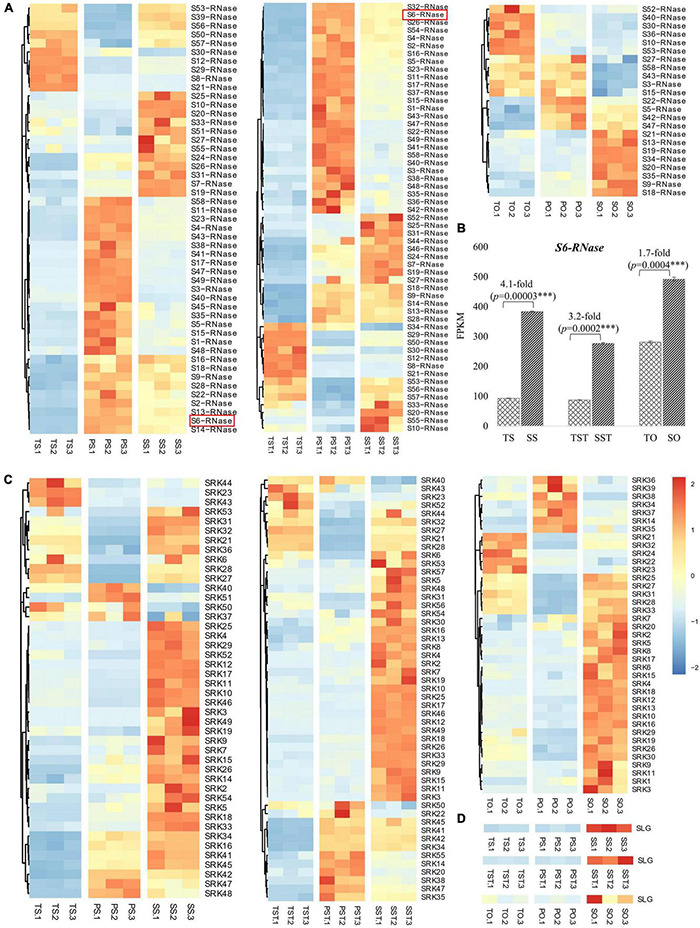
Expression changes of S-locus genes in floral tissues of Seedless palm trees compared with Tenera and Pisifera. T, Tenera; P, Pisifera; S, seedless; S, stigma; ST, style; O, ovary. **(A)** Heatmap showing the expressions patterns of S-RNases. **(B)** Expression changes of *S6-RNase* which increased greater than threefold in stigma (S) and style (ST) of Seedless palm trees compared with Tenera. **(C)** Heatmap showing the expressions patterns of SRK genes. **(D)** Heatmap showing the expressions patterns of SLG gene.

**FIGURE 7:**
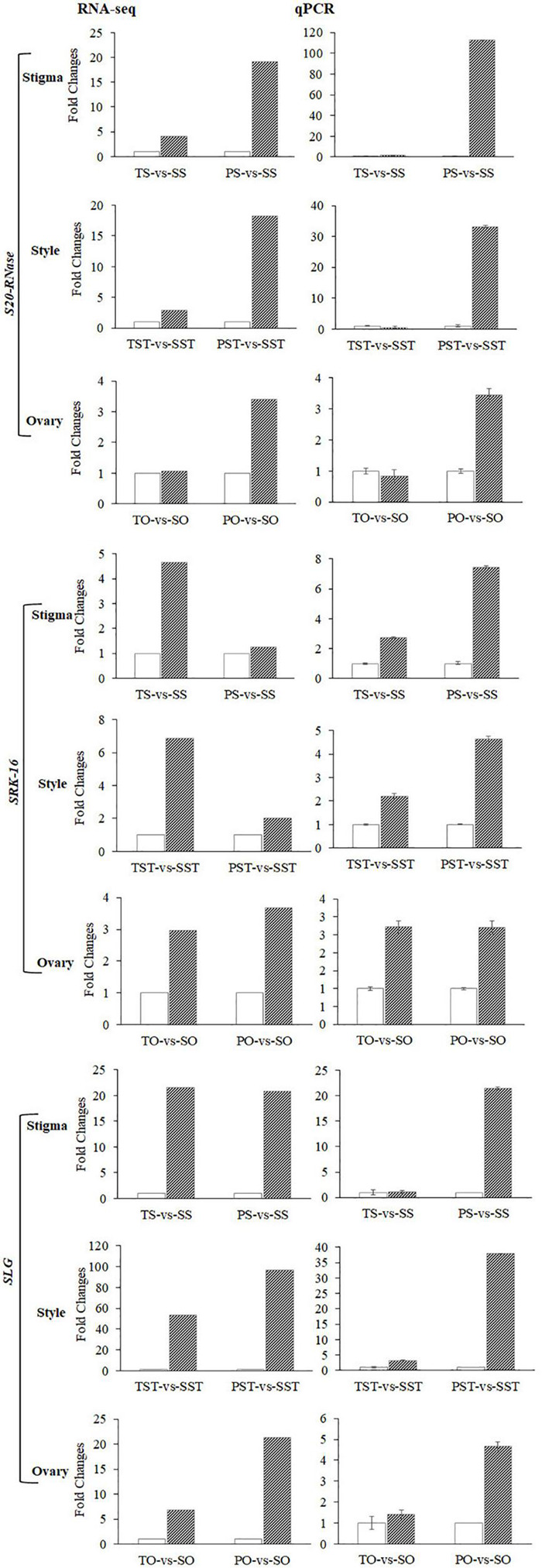
Expression changes of *S20-RNase*, *SRK-16*, and *SLG* genes revealed by RNA-seq and RT-qPCR.

To identify S-RNase-related genes, previously reported six known S-RNases were downloaded from NCBI (HE805271.1 and AJ315593.1 from *Antirrhinum*; D63887.1 and AB568389.1 from *Solanaceae*; and FJ543097.1 and AF327223.1 from *Rosaceae*) to detect sequence similarity with DEGs in Tenera-vs-Seedless and Pisifera-vs-Seedless. Transcriptome analysis enabled the identification of DEGs highly homologous (>75% similarity) to known S-RNases. Results showed that identified S-RNases were more abundantly expressed in stigma and style than in the ovary (Figure 6A). Furthermore, *S6-RNase* is highly expressed in stigma (383.57 FPKM) and style (276.97 FPKM) of Seedless palm trees which is greater than threefold higher than Tenera (Figure 6B). Similarly, more G-type lectin S-receptor-like serine/threonine-protein kinase (SRK) genes were expressed in stigma and style than in the ovary (Figure 6C). One gene encoding S-locus-specific glycoprotein (SLG) was expressed in Seedless palm trees compared with Tenera and Pisifera (Figure 6D). Altogether, results showed that S-locus genes, in particular S-RNases and SRK, were more abundantly expressed in stigma and style than in the ovary (Figure 6). We further conducted a comparative analysis of the expression data obtained by RNA-seq with RT-qPCR in stigma, style, and ovary tissues to corroborate the reliability of the RNA-seq data. High consistency was verified between RNA-seq and RT-qPCR results, confirming that the expression data obtained by RNA-seq is reliable (Figure 7).

### Analysis of MADS-Box TFs

**FIGURE 8:**
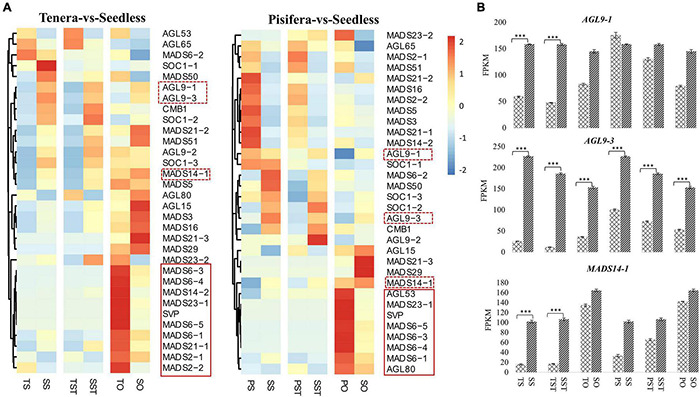
Expression patterns of MADS-box genes. **(A)** Heatmap showing the expression patterns of MADS-box genes in Tenera-vs-Seedless and Pisifera-vs-Seedless. Red dotted boxes represent the genes that were highly expressed (>100 FPKM) in Seedless palm trees and showed increased expressions compared with Tenera and Pisifera. Red solid line boxes represent genes that were down-regulated in the ovary of Seedless palm trees compared with Tenera and Pisifera. **(B)** Expression patterns of highly expressed MADS-box genes. T, Tenera; P, Pisifera; S, Seedless; S, stigma; ST, style; O, ovary. Asterisks (^***^P ≤ 0.001) indicate a significant difference according to the Student’s *t*-test.

MADS-box TFs are reported to have a role in pollen fertilization, ovule, and seed development (Wei et al., 2019). Previous research on tomato had shown that mutation in some MADS-box genes allows seedless fruit development (Klap et al., 2017; Takisawa et al., 2018; Molesini et al., 2020). To gain insight into the function of MADS-box genes in oil palm seedless phenotype formation, we analyzed their expression patterns that changed in Seedless palm trees compared to Tenera and Pisifera. Results showed that a total of 32 MADS-box genes were expressed in Tenera-vs-Seedless and Pisifera-vs-Seedless. Of them, more genes were up-regulated in TS-vs-SS and TST-vs-SST (Figure 8A). Notably, *AGL9-1*, *AGL9-3*, and *MADS14-1* genes were found to express high levels (>100 FPKM) in Seedless palm trees and showed increased expressions compared with Tenera and Pisifera (Figure 8B). This suggests the important contribution of these genes to seedless phenotype formation in oil palm.

### Analysis of Auxin Signaling Related Genes

**FIGURE 9:**
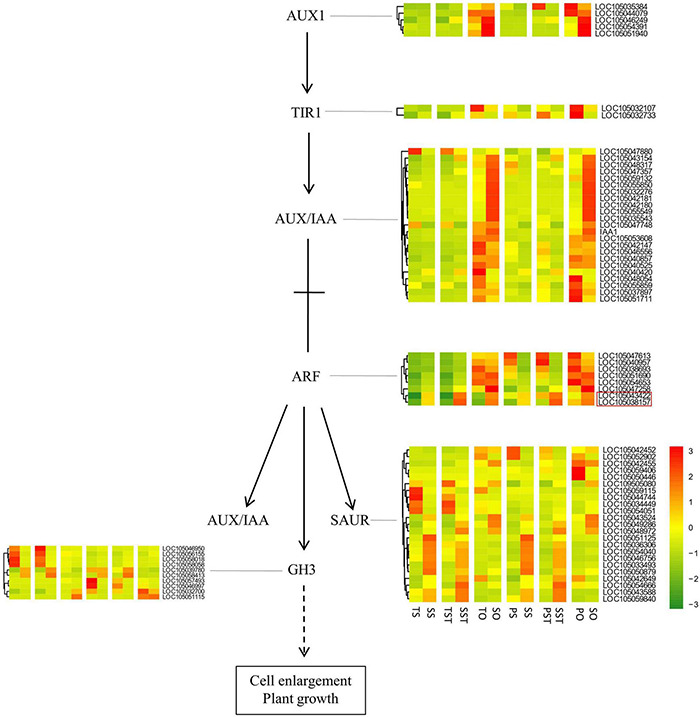
Analysis of the genes in auxin signaling pathway. Red box indicates ARF genes up-regulated in Seedless palm trees (S) compared with Tenera (T) and Pisifera (P).

A recent study by Kacprzyk et al. (2021) suggested the role of interplay between PCD and auxin signaling in the plant developmental process. In our study, analysis of the expression changes of auxin signaling genes revealed that auxin response factor (ARF) genes [LOC105043422 (ARF15) and LOC105038157 (ARF1)] were up-regulated in Seedless palm trees (S) compared with Tenera (T) and Pisifera (P) (Figure 9). It might be possible that crosstalk between auxin signaling and PCD plays a role in inducing seedless phenotype formation. However, because of the complex interactions among different plant hormones and PCD regulation, further detailed investigations are still needed.

### Gene Co-expression Network Analysis

**FIGURE 10:**
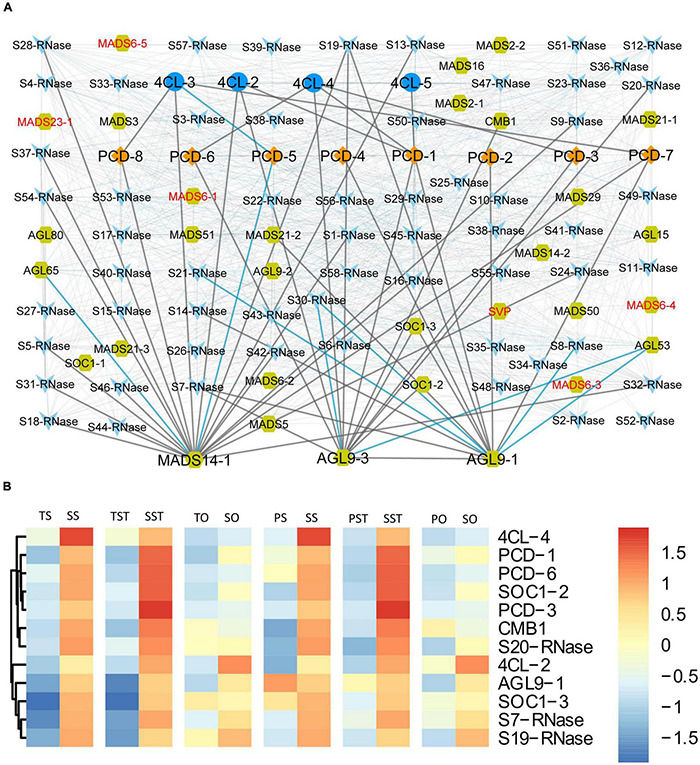
Analysis of potential cooperation among PCD genes (PCD1–PCD8) and three groups of PCD-related genes: 4CL, S-RNase, and MADS-box **(A)** Co-expression network of genes. Red texts indicate MADS-box genes which were specifically down-regulated in the ovary of Seedless palm trees compared with Tenera and Pisifera. Gray edges linking nodes represent positive correlation while blue edges represent a negative correlation. Nodes: PCD, diamond; 4CL, ellipse; S-RNase, V-shaped; MADS-box, hexagon. **(B)** Heatmap showing the expression patterns of genes positively correlated with *AGL9-3*.

In rice, *OsMADS29* affects the degeneration of cells by regulating PCD-related genes (Yang et al., 2012). Despite many reports on MADS-box TFs regulating flower and seed development, little is known regarding the cooperation between MADS-box TFs and PCD in oil palm. To address this, co-expression network among PCD genes (*PCD1*–*PCD8*) and three groups of PCD-related genes (4CL, S-RNase, and MADS-box) was constructed. Here, we used the term PCD-related genes formally for the genes that have previously been reported to play a role in inducing PCD. Significantly correlated pairs (*p* < 0.01) were visualized using Cytoscape (Shannon et al., 2003). The resulting network shows that 4CL genes were positively correlated with PCD genes which were up-regulated in Seedless palm trees (*PCD-1*, *PCD-3*, *PCD-6*, *PCD-7*, and *PCD-8*) (Figure 10A). Notably, most genes that were highly up-regulated (greater than twofold) in the tested floral tissues of Seedless palm trees, especially in the stigma and style, were positively correlated with *AGL9-3* (Figure 10B). Furthermore, MADS-box genes which were specifically down-regulated in the ovary of Seedless palm trees were directly or indirectly correlated with PCD genes and PCD-related genes (Figure 10A), suggesting the possible interaction among these genes.

**FIGURE 11:**
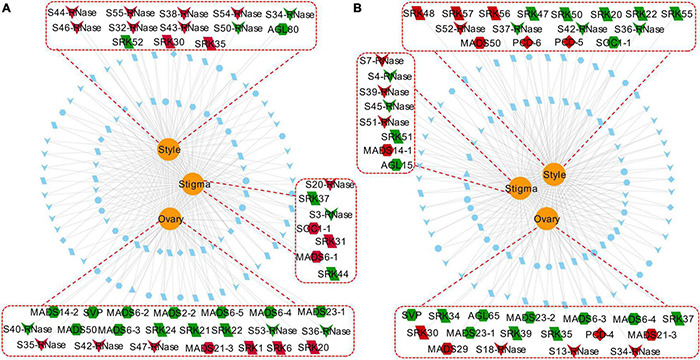
Network showing commonly and specifically expressed PCD genes (PCD1–PCD8) and three groups of PCD-related genes (4CL, S-RNase, and MADS-box) in different floral tissues (stigma, style, and ovary). Red dotted lines and rectangles indicate genes specifically expressed in the corresponding tissue. Edges represent target genes by the corresponding tissue, in which red nodes indicate up-regulated genes while green nodes indicate down-regulated genes. Blue nodes represent genes expressed in common across tissues. **(A)** Tenera-vs-Seedless. **(B)** Pisifera-vs-Seedless. Nodes: PCD, diamond; 4CL, ellipse; S-RNase, V-shaped; MADS-box, hexagon; SLG, triangle; SRK, parallelogram.

We next examined the expressions of PCD and PCD-related genes that were specifically expressed in different floral tissues (stigma, style, and ovary) of Tenera-vs-Seedless and Pisifera-vs-Seedless. We observed that most genes specifically expressed in the ovary, especially MADS-box TFs, were down-regulated in Seedless palm trees compared with Tenera and Pisifera (Figure 11).

### Seedless Phenotype Identification by Simple Sequence Repeats Markers

**FIGURE 12:**
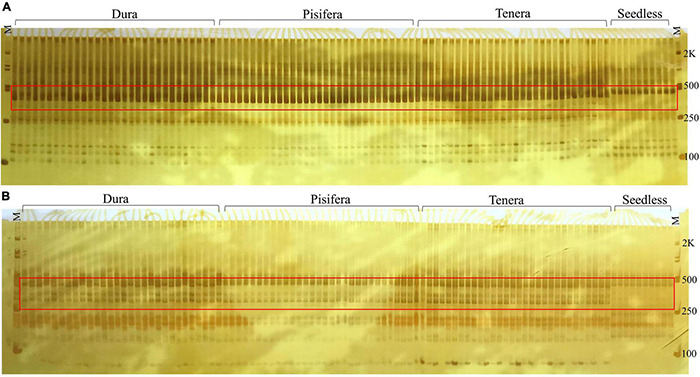
Markers **(A)** S41 and **(B)** S44 showed distinction between seedless and seeded (Dura, Pisifera, and Tenera) palm trees, where M represents 100-bp marker.

In this study, we used S-RNase specific primers to identify potential markers that can be used for the selection of seedless plants in oil palm. Out of the 20 SSR primers, two primers (S41 and S44) were found to be polymorphic for Seedless palm trees. Further verification of the primers with 100 individuals showed the accuracy of these markers in determining seedless phenotype (Figure 12).

## Discussion

The production of oil palm seedless fruits has attracted attention since long because of its high oil-yielding capacity (Somyong et al., 2018; Shi et al., 2019; Romero et al., 2021). While reports with regard to seedless fruits have been scattered in a number of plant species (Goetz et al., 2007; Lora et al., 2011; Chen et al., 2017; Klap et al., 2017; Malabarba et al., 2017; Takisawa et al., 2018; Zhang et al., 2018; Liang et al., 2020; Molesini et al., 2020; di Rienzo et al., 2021; Wang et al., 2021a,b), the regulatory mechanism controlling seedless trait in oil palm is much less well understood. Here, we performed self-, cross-, and non-pollinations to elucidate the possible mechanism of inducing seedless phenotype in oil palm. All self- and cross-pollinations resulted in about 30% seedless fruits while non-pollinations resulted in no fruit, suggesting incomplete self and outcrossing incompatibility in seedless trees. As a fact, the majority of fruits from the top and out layer of the bunches were seedlessness, instead of all fruits. This could be due to environmental factors, for example, enough sunlight or too much pollens at out layer female flowers that resulted in excessive competition and double fertilization failure.

Results from the *in vitro* pollen germination assays show that pollens from seedless pistil were germinated normally. It was reported that boron deficiency affects pollen germination, pollen tube growth, fruit set, and seed formation, thereby inhibiting reproductive growth (Alva et al., 2015). To further determine if boron treatment can affect seedless phenotype formation in oil palm, different concentrations of boron were applied on opened female inflorescences three times (once per day) and compared with untreated control. Similar to untreated control, boron-treated plants produced seedless fruits, indicating that boron treatment has no effect on seedless phenotype formation. This implies that seedless fruits development in oil palm does not rely on pollination and boron, but may be driven by genetic factors.

Thus, with the aim of elucidating mechanism and genetic factors underlying seedlessness in oil palm, we performed transcriptional analysis to compare the transcriptomes of seedless and seeded (Tenera and Pisifrea) oil palm using floral tissues (stigma, style, and ovary). Analysis of DEGs revealed more DEGs in stigma and style than in the ovary. Furthermore, correlation analysis also showed that stigma and style have similar transcriptome profiles. Given that the identification of metabolic pathways would be beneficial to crop improvement (Zhang et al., 2021), we further revealed the metabolic pathways enriched between seeded and seedless oil palm. Pathway analysis showed that phenylpropanoid biosynthesis pathway, which has previously been reported to play a leading role in male sterility of citrus seedless mutant (Zhang et al., 2018), was significantly enriched between seeded and Seedless palm trees in all the tested floral tissues. Phenylpropanoid biosynthesis pathway plays an important role in the development of floral organs (Zhang et al., 2018). Consistent with this fact, the present study observed that 4CL, which is involved in phenylpropanoid biosynthesis pathway, was found to be enriched in pollen development (GO:0009555) and pollen wall assembly (GO:0010208) of GO biological process.

Previous studies revealed that the absence of seed formation is associated with failure in the development of ovule where fertilization takes place. In seedless pear, PCD occurred after pollination results in unfertilized ovule senescence (Skinner et al., 2004; Lora et al., 2011; Wang et al., 2021a). This suggests that PCD mechanism plays a vital role in inducing seedless fruits. In rice, up-regulation of lignin biosynthesis genes, including 4CL, resulted in an increased content of lignin and led to activate PCD (Chen et al., 2020). It was also reported that overexpression of *OsAAE3*, which encodes a 4CL like protein, can lead to PCD, which contributed to the repressing of floral development and decreased fertility rate of the anther (Liu et al., 2017a). In the present study, we found that most 4CL genes were significantly up-regulated (^***^P ≤ 0.001) in seedless compared with seeded (Tenera and Pisifera) palm trees. Previous study in seedless pear revealed that PCD mechanism could lead to ovule abortion (Wang et al., 2021b). In our current study, most PCD genes were found to be up-regulated in seedless pistil. Apparently, most genes were highly expressed in stigma and style while they were weakly expressed in the ovary. This suggests the important contribution of these genes to oil palm seedless phenotype formation.

Self-incompatibility, also known as self-sterility, is a mechanism that rejects their self-pollen and inhibits seed formation in the absence of cross-pollination (Montalt et al., 2021). Molecular studies revealed that genes within the S-locus, including S-RNase, SRK, and SLG, regulate the SI system (Kachroo et al., 2002; Wang et al., 2009; Liang et al., 2020). Previous works have reported that S-RNase mediates the PCD mechanism by catalyzing the degradation of RNA (Wang et al., 2009; Claessen et al., 2019). Although ribonuclease activity of S-RNases is essential for SI response, it is worth noting that S-RNase also functions as the regulator of PCD (Takayama and Isogai, 2005; Franceschi et al., 2012; Muñoz-Sanz et al., 2020). When pollens fall on stigma, S-RNase is produced and they enter into the stigma surface. Thereafter, they degrade the RNA coding enzyme for pollen tube growth which results in death of the pollen tube (Narayanapur et al., 2018). It has been proposed that SRK and SLG cooperatively function in SI response of Brassica (Takasaki et al., 2000). In the present study, we have identified S-RNases, which had strong homology to previously reported known S-RNase sequences from species with S-RNase-based SI (Liang et al., 2020) as well as SRK and SLG. Thereafter, we analyzed their expression patterns to evaluate their roles in oil palm seedlessness. Results showed that these S-locus genes were more abundantly expressed in the stigma and style than in the ovary. In Tenera-vs-Seedless, more S-RNases were up-regulated in Seedless palm trees. Of them, *S6-RNase* is highly expressed in stigma (383.57 FPKM) and style (276.97 FPKM) of Seedless palm trees which is greater than threefold higher than Tenera. In contrast, the number of up-regulated S-RNases were slightly lower than down-regulated S-RNases in Pisifera-vs-Seedless. This may be due to the fact that S-RNases are female determinant and Pisifera palms are female sterile (Singh et al., 2013; Liang et al., 2020). Nonetheless, further research is still needed to clarify this idea.

It has been proposed that different fruit forms in oil palm are controlled by *SHELL*, a type II MADS-box gene that controls ovule identity and seed development in Arabidopsis (Singh et al., 2013, 2020). More so, MADS-box genes have a role in pollen fertilization and the development of seedless fruits (Klap et al., 2017; Takisawa et al., 2018; Tang et al., 2019; Wei et al., 2019; Molesini et al., 2020). A previous study (Ocarez and Mejía, 2016) reported that MADS-box gene *AGL11* plays a major role in triggering the seedless phenotype. Thus, we further focused on the expression changes of MADS-box genes and observed that the majority of genes showed increased expression in the stigma and style of Seedless palm trees compared with Tenera. Notably, *AGL9-1*, *AGL9-3*, and *MADS14-1* genes were highly expressed in Seedless palm trees and showed higher expressions than Tenera and Pisifera. This suggests the important contribution of these genes to seedless phenotype in oil palm. In rice, *OsMADS29* affects the degeneration of cells by regulating PCD-related genes (Yang et al., 2012). Although there are many reports on MADS-box TFs regulating flower and seed development, little is known regarding the cooperation between MADS-box TFs and PCD in oil palm.

Accumulating evidence (Liu et al., 2017b; Ding et al., 2020; Zhang et al., 2021) suggest that studies on gene interaction networks are very useful for investigating the mechanism of gene function. Hence, co-expression network among PCD genes (PCD1–PCD8) and three groups of PCD-related genes (4CL, S-RNase, and MADS-box) were constructed. The resulting network showed that genes encoding 4CL were positively and significantly correlated with PCD genes that were up-regulated in Seedless palm trees. Consistent with previous studies (Liu et al., 2017a; Chen et al., 2020), our results suggest that 4CL genes could trigger PCD *via* activating the expression of PCD genes. Notably, most genes that were highly up-regulated (greater than twofold change) in the tested tissues of Seedless palm trees, especially in stigma and style, were positively and significantly correlated with *AGL9-3*. Our results suggested that MADS-box genes, in particular *AGAMOUS-Like* 9 (*AGL9-3*), contributed to the positive role in triggering PCD in oil palm by their upregulation. Furthermore, MADS-box genes which were specifically down-regulated in the ovary of Seedless palm trees were directly or indirectly correlated with PCD genes and PCD-related genes, suggesting the possible interaction among these genes.

We next analyzed PCD genes and PCD-related genes that were specifically expressed in different floral tissues (stigma, style, and ovary) of Tenera-vs-Seedless and Pisifera-vs-Seedless. We observed that most genes specifically expressed in the ovary, especially MADS-box TFs, were down-regulated in Seedless palm trees compared with Tenera and Pisifera. In accordance with previous studies (Klap et al., 2017; Takisawa et al., 2018; Molesini et al., 2020), our results suggest the significant role of MADS-box genes in inducing seedless fruits.

Additionally, due to the polymorphic nature of S-locus (Hua et al., 2008), SSR markers were developed from S-RNase-related gene to facilitate the selection of seedless plants. Our study found that markers S41 and S44 showed a distinction between seeded and seedless plants and would be very useful for the early identification of seedless phenotype.

**FIGURE 13:**
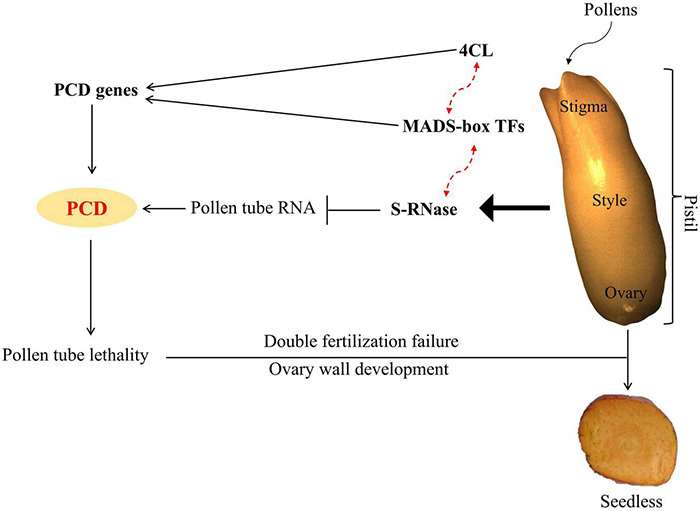
Model depicting the possible mechanism for inducing seedless traits in oil palm. Solid lines indicate where the function of genes is known, while red dotted lines indicate the predicted function from our study.

In conclusion, results suggested that up-regulation of 4CL and MADS-box TFs activated the expression of PCD genes; on the other hand, S-RNase resulted in pollen tube RNA degradation and triggered PCD. Thus, PCD leads to pollen tube lethality, resulting in double fertilization failure and seedless phenotype (Figure 13).

## Data Availability Statement

The RNA-seq data for this study can be found in the National Genomics Data Center-Genome Sequence Archive (GSA accession: XCRA005546) (https://bigd.big.ac.cn/gsa/browse/CRA005546).

## Author Contributions

YW designed and supervised the experiments. YMH, PS, and DZ performed the data analysis and wrote the manuscript. PS and ZL prepared the samples. YX, YY, XL, and YW reviewed the manuscript. YMH, PS, DZ, and YW designed the primers and revised the manuscript. All authors read and approved the final manuscript.

## Conflict of Interest

The authors declare that the research was conducted in the absence of any commercial or financial relationships that could be construed as a potential conflict of interest.

## Publisher’s Note

All claims expressed in this article are solely those of the authors and do not necessarily represent those of their affiliated organizations, or those of the publisher, the editors and the reviewers. Any product that may be evaluated in this article, or claim that may be made by its manufacturer, is not guaranteed or endorsed by the publisher.

## Footnotes

1
https://bioinfo.inf.ufg.br/websat/
