# Supplementary figures and images for: Programmed Cell Death May Be Involved in the Seedless Phenotype Formation of Oil Palm
Source: Front Plant Sci. 2022 Mar 23;13:832017. doi: 10.3389/fpls.2022.832017 (PMC8984474; doi:10.3389/fpls.2022.832017)

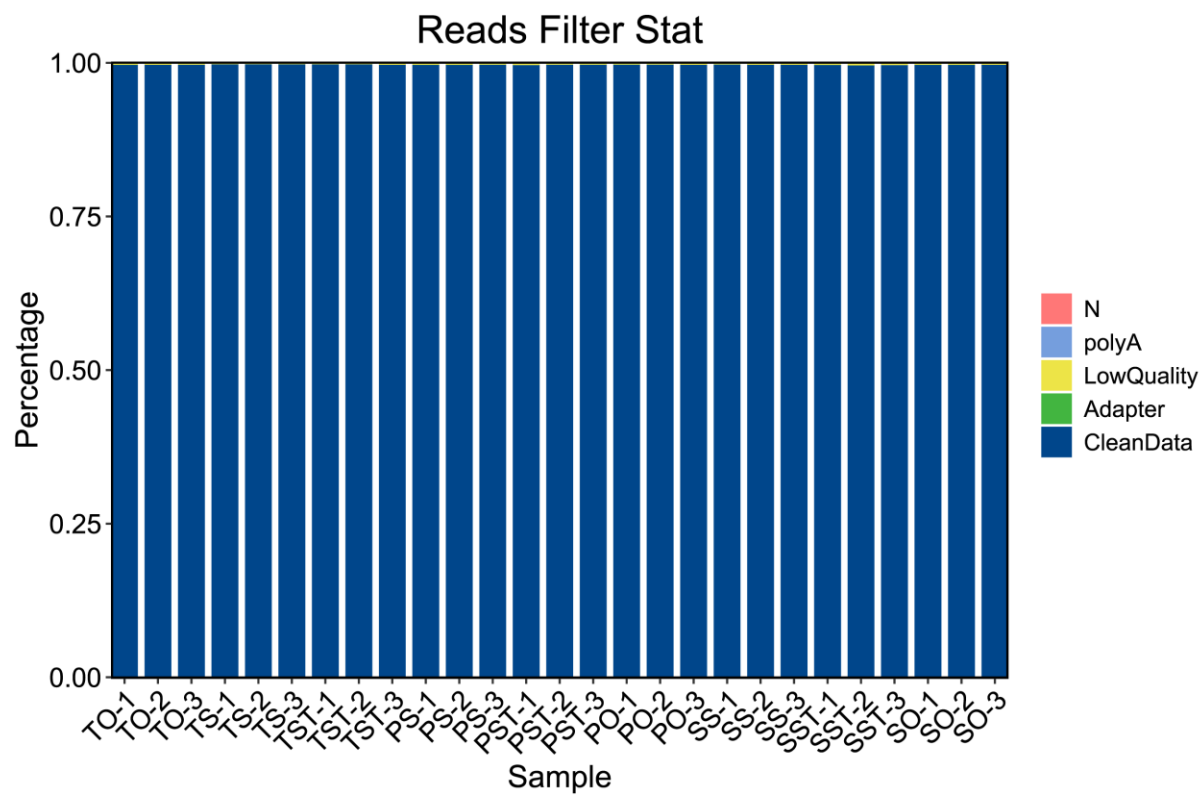

**Supplementary Figure 1.** Quality assessment of reads. T, Tenera; P, Pisifera; O, Ovary; S, Stigma; ST, Style.

Supplement: Supplementary file 6 [file Image_1.PDF]
