# Supplementary material for: Programmed Cell Death May Be Involved in the Seedless Phenotype Formation of Oil Palm
Source: Front Plant Sci. 2022 Mar 23;13:832017. doi: 10.3389/fpls.2022.832017 (PMC8984474; doi:10.3389/fpls.2022.832017)
Supplement: Supplementary file 7 [file Image_2.PDF]

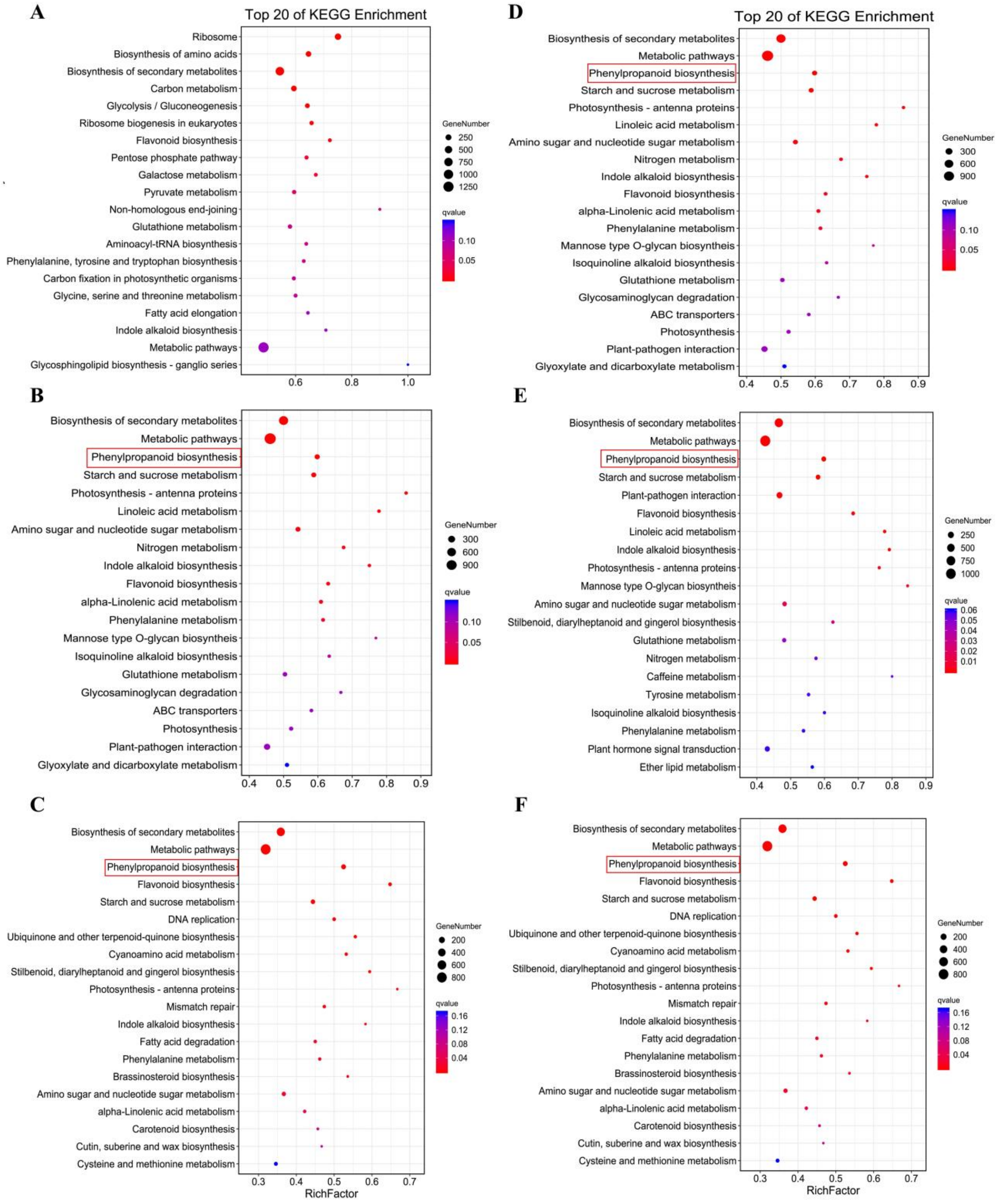

Supplementary Figure 2. Top 20 KEGG pathways enriched between groups. **(A)** TS-vs-SS. **(B)** TST-vs-SST. **(C)** TO-vs-SO. **(D)** PS-vs-SS. **(E)** PST-vs-SST. **(F)** PO-vs-SO. The size of the bubble indicates the number of genes. T, Tenera; P, Pisifera; S, Stigma; ST, Style; O, Ovary.
